# Rickettsial Illnesses as Important Causes of Febrile Illness in Chittagong, Bangladesh

**DOI:** 10.3201/eid2404.170190

**Published:** 2018-04

**Authors:** Hugh W. Kingston, Mosharraf Hossain, Stije Leopold, Tippawan Anantatat, Ampai Tanganuchitcharnchai, Ipsita Sinha, Katherine Plewes, Richard J. Maude, M.A. Hassan Chowdhury, Sujat Paul, Rabiul Alam Mohammed Erfan Uddin, Mohammed Abu Naser Siddiqui, Abu Shahed Zahed, Abdullah Abu Sayeed, Mohammed Habibur Rahman, Anupam Barua, Mohammed Jasim Uddin, Mohammed Abdus Sattar, Arjen M. Dondorp, Stuart D. Blacksell, Nicholas P.J. Day, Aniruddha Ghose, Amir Hossain, Daniel H. Paris

**Affiliations:** Charles Darwin University, Casuarina, Northern Territory, Australia (H.W. Kingston);; Mahidol University, Bangkok, Thailand (H.W. Kingston, S. Leopold, T. Anantatat, A. Tanganuchitcharnchai, I. Sinha, K. Plewes, R.J. Maude, A.M. Dondorp, S.D. Blacksell, N.P.J. Day, D.H. Paris);; Chittagong Medical College Hospital, Chittagong, Bangladesh (M. Hossain, M.A.H. Chowdhury, S. Paul, R.A.M.E. Uddin, M.A.N. Siddiqui, A.S. Zahed, A.A. Sayeed, M.H. Rahman, A. Barua, M.J. Uddin, M.A. Sattar, A. Ghose, A. Hossain);; Oxford University, Oxfordshire, UK (I. Sinha, R.J. Maude, A.M. Dondorp, S.D. Blacksell, N.P.J. Day, D.H. Paris);; Harvard University, Boston, Massachusetts, USA (R.J. Maude);; Swiss Tropical and Public Health Institute, Basel, Switzerland (D.H. Paris); University of Basel, Basel (D.H. Paris)

**Keywords:** Bangladesh, scrub typhus, murine typhus, rickettsia, Orientia tsutsugamushi, Rickettsia typhi, Rickettsia felis, undifferentiated febrile illness, rickettsial disease, vector-borne diseases, bacteria

## Abstract

We conducted a yearlong prospective study of febrile patients admitted to a tertiary referral hospital in Chittagong, Bangladesh, to assess the proportion of patients with rickettsial illnesses and identify the causative pathogens, strain genotypes, and associated seasonality patterns. We diagnosed scrub typhus in 16.8% (70/416) and murine typhus in 5.8% (24/416) of patients; 2 patients had infections attributable to undifferentiated *Rickettsia* spp. and 2 had DNA sequence–confirmed *R. felis* infection. *Orientia tsutsugamushi* genotypes included Karp, Gilliam, Kato, and TA763-like strains, with a prominence of Karp-like strains. Scrub typhus admissions peaked in a biphasic pattern before and after the rainy season, whereas murine typhus more frequently occurred before the rainy season. Death occurred in 4% (18/416) of cases; case-fatality rates were 4% each for scrub typhus (3/70) and murine typhus (1/28). Overall, 23.1% (96/416) of patients had evidence of treatable rickettsial illnesses, providing important evidence toward optimizing empirical treatment strategies.

## Abstract 2

Scrub and murine typhus are common, treatable causes of undifferentiated febrile illnesses in hospitalized patients.

The prevalence, seasonality, and genotypes of tropical rickettsial illnesses in Bangladesh remain unknown. Scrub and murine typhus typically present as undifferentiated febrile illnesses and are insensitive to penicillins and cephalosporins, common antimicrobial drugs used empirically (*1*). Rickettsial illnesses are increasingly recognized as important causes of fever in adjacent countries in the region, including Thailand, Laos, and India (*2*–*5*). Therefore, determining whether rickettsial infections are widespread in Bangladesh, a low-income country with a population of ≈160 million, is of urgent public health interest. 

Cross-sectional seroprevalence studies suggest that exposure to scrub typhus and murine typhus is common (*6*). In addition, given the substantial proportion of febrile patients from north-central Bangladesh with confirmed *R. felis* infection (*7*), further investigations regarding the clinical relevance of this pathogen are warranted.

Identification and characterization of circulating pathogens and strains is crucial for the development of diagnostics and vaccines (*8*). Strong seasonal trends might guide differential diagnostic considerations. Therefore, we conducted a year-long prospective study of febrile patients admitted to a tertiary referral hospital with a wide catchment area in southeast Bangladesh, aiming to assess the proportion of patients with rickettsial illnesses, identify the causative pathogens, strain genotypes, and the associated seasonality patterns.

## Materials and Methods

We conducted our study at Chittagong Medical College Hospital (CMCH), Chittagong, Bangladesh, during August 2014–September 2015. We enrolled into the study all consenting patients >12 years old who were admitted with a febrile illness to the medical wards and referred to the hospital’s malaria screening service with a history of fever for <3 weeks. Written informed consent was provided by all patients before inclusion in the study or by their relatives if the patient lacked capacity for providing consent. The study was approved by Chittagong Medical College ethics committee in Bangladesh and the Oxford Tropical Research Ethics Committee, United Kingdom.

We collected admission blood samples and convalescent-phase blood samples (taken 7–14 days apart, where possible) into EDTA tubes and separated the samples into packed cells and plasma before storage at −30°C. We subjected packed cell admission samples of all patients to DNA extraction for real-time PCR screening by using the 47-kDa *htra* gene–based assay for *Orientia* spp. and the 17-kDa gene–based assay for *Rickettsia* spp. We subsequently confirmed positive results by nested PCR (nPCR) assays with product sequencing. For *Orientia* spp., we targeted the 56-kDa and 47-kDa gene targets. For *Rickettsia* spp., we targeted 17-kDa and performed *ompB* real-time PCR and *gltA* nPCR, as previously described (*9*,*10*). 

We aligned the resulting DNA sequences for construction of phylogenetic trees by using CLC Sequence Viewer 7.0.2 (http://www.clcbio.com). For serologic testing, all patient plasma samples underwent indirect immunofluorescence assays (IFA) by using slides from the Australian Rickettsial Reference Laboratory coated with *O. tsutsugamushi* (strains Karp, Kato, and Gilliam) for scrub typhus or *R. typhi* (Wilmington strain) for murine typhus. We used the following stringent diagnostic positivity criteria for scrub and murine typhus: a baseline IgM titer of >3,200 or a 4-fold rise to >3,200. In the absence of regional serologic positivity criteria for Bangladesh, we selected these cutoffs on the basis of experience from an area where scrub typhus is highly endemic (*11*). In suspected cases of *R. felis* infection, we conducted IFA by using dedicated IFA slides from the Australian Rickettsial Reference Laboratory. We conducted statistical analyses by using Stata 14 (StataCorp LLC, College Station, TX, USA). 

## Results

We screened 901 patients for enrollment; 794 patients met the enrollment criteria and, of these, 416 gave consent and were enrolled into the study. A total of 414 patient admission samples were available for PCR and 415 for serologic testing; 256/416 (62%) patients were followed up to provide paired samples. Of the enrolled patients, 16.8% (70/416) had a robust diagnosis of scrub typhus and 5.8% (24/416) of murine typhus by PCR, serologic testing, or both (Table). Two patients had evidence of both typhus group and scrub typhus infection, and 2 patients were found to have undifferentiated *Rickettsia* spp. infections. Samples from 2 patients were positive for *R. felis* (1 patient’s blood was positive for *R. felis* by 17kDa nPCR and sequencing; the second patient was positive for *O. tsutsugamushi* by 47kDa and 56kDa nPCR and 56kDa gene sequencing from blood and eschar, but also had a superficial eschar swab positive for *R. felis* by 17kDa nPCR and sequencing, suggestive of scrub typhus infection with possible skin carriage of *R. felis* on the skin or eschar). Both of these patients with PCR evidence of *R. felis* infection were serologically negative (titer <1:10) by *R. felis*–specific IFA. In this prospective cohort of hospitalized febrile patients, 23.1% (96/416) of the fevers were attributable to rickettsial illnesses.

**Table:** Results of PCR and serologic tests for rickettsial illness among 416 patients, Chittagong Medical College Hospital, Chittagong, Bangladesh, August 2014–September 2015*

Organism and test type	No. positive/no. tested (%)
*Orientia tsutsugamushi*	70/416 (16.8)
Blood PCR, rPCR 47-kDa positive	45/414 (10.9)
nPCR 47 kDa positive	45/45 (100)
nPCR 56 kDa positive	45/45 (100)
Eschar swab, rPCR 47 kDa and n56kDa positive; crust (n = 1), swab (n = 3)	3/416 (0.7)
Indirect immunofluorescence assay	57/415 (13.7)
Admission titer >3,200	54/415 (13.0)
4-fold rise to >3,200	31/255 (12.1)
PCR+ and serology+, 32/70 (45.7% of ST positives)	32/413 (7.7)
PCR+ and serology–, 13/70 (18.6% ST positives)	13/413 (3.1)
PCR– and serology+, 25/70 (35.7% of ST positives)	25/413 (6.0)
*Rickettsia* spp.	29/416 (7.0)
Blood PCR, rPCR 17 kDa positive	23/414 (5.6)
nPCR 17 kDa positive	16/23 (69.6)
*Rickettsia typhi*, 24/29 (83.0%) of *Rickettsia* spp.	24/416 (5.8)
Blood PCR	17/414 (4.1)
rPCR *OmpB* positive	12/414 (2.9)
nPCR 17-kDa sequencing	15/16 (93.8)
Indirect immunofluorescence assay	15/415 (3.6)
Admission titer >3,200	11/415 (2.7)
4-fold rise to >3,200	5/255 (2.0)
PCR+ and serology+, 8/24 (33.3% of MT positives)	8/413 (1.9)
PCR+ and serology–, 9/24 (37.5% of MT positives)	9/413 (2.2)
PCR– and serology+, 7/24 (29.1% of MT positives)	7/413 (1.7)
Undifferentiated *Rickettsia* spp., 3/29 (10.3% of *Rickettsia* spp.)	3/416 (0.7)
rPCR 17-kDa positive, *ompB* negative	3/416 (0.7)
nPCR 17-kDa negative, *gltA* negative	3/416 (0.7)
MT serology negative	3/416 (0.7)
* Rickettsia felis*	2/416 (0.5)
Blood PCR, 17-kDa rPCR and nPCR	1/416 (0.2)
Eschar swab, 17-kDa rPCR and nPCR	1/416 (0.2)
All rickettsial illnesses†	96/416 (23.1)

*MT, murine typhus; nPCR, nested PCR; rPCR, real-time PCR; ST, scrub typhus. †Twenty-nine patients had evidence of *Rickettsia* spp. Infection; 70 had evidence of *O. tsutsugamushi* infection. Because 2 case-patients had mixed blood *O. tsutsugamushi* and *Rickettsia* spp. infections and 1 case-patient with *O. tsutsugamushi* infection in addition to an eschar-positive swab for *R. felis*, the total number of rickettsial illness cases was 96.

**Figure 1:**
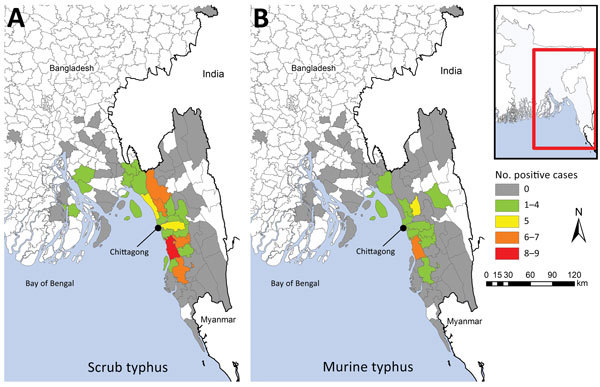
Geographic distribution of scrub typhus (A) and murine typhus (B) cases, Chittagong, Bangladesh, August 2014–September 2015. Inset shows location of enlarged area (red box).

We plotted the geographic distribution of scrub typhus and murine typhus cases (Figure 1), reflecting the catchment area of the hospital in southeast Bangladesh. The case-patient with blood PCR positivity for *R. felis* was from urban Chittagong, whereas the case-patient with eschar swab positivity for *R. felis* and blood positivity for *O. tsutsugamushi* came from a rural area. Only 12% (8/67) of patients with scrub typhus came from urban areas, compared with 27% (84/310) of those without evidence of rickettsia (p = 0.009).

**Figure 2:**
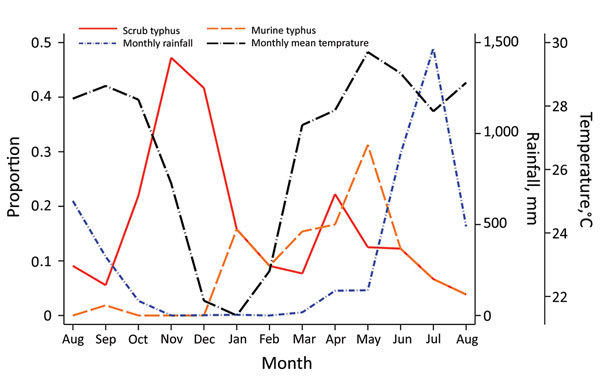
Seasonality of scrub typhus and murine typhus–related hospital admissions, Chittagong Medical College Hospital, Chittagong, Bangladesh, August 2014–September 2015. We observed a biphasic pattern in scrub typhus, with an increase of cases in the cooler months and a smaller peak before the rainy season.

We observed 2 peaks of scrub typhus patient admissions during the study period, 1 in the cooler months of November and December, when 32/72 (44%) patients were found to be positive for scrub typhus, and the other before the rainy season in April and May, with 6/34 (18%) of cases. Murine typhus cases peaked in the months before the rainy season (Figure 2).

Among all patients with rickettsial illnesses, the most common complaints were headache, anorexia, and myalgia, whereas rash was only detected in 6/96 (6%) of patients. In scrub typhus cases, an eschar was found in only 3/70 (4%) of patients, despite training and use of a dedicated checklist for identifying eschars included in the clinical record form. The blood PCR–positive *R. felis* case (SW148) had a different manifestation than other rickettsioses: an itchy vesicular–petechial rash similar to spotted fever group rickettsioses that started periorally and distributed to the trunk and extremities. The patient reported contact with rats in the 3 weeks before illness onset. Overall, 4 deaths were attributable to typhus during this study (3/70 [4.3%] of the patients with scrub typhus and 1/28 [3.6%] patient in the murine typhus group). Of the rickettsia-negative case-patients, 14/320 (4.4%) died. All 3 of the patients with scrub typhus who died had clinical evidence of meningoencephalitis. At study enrollment, the first patient had a Glasgow Coma Scale of 7, severe respiratory distress, new atrial fibrillation with a rapid ventricular response, and hypotension with cold peripheries, consistent with septic shock. The second patient had impaired consciousness, meningism, dyspnea, chest pain, and cough. The third patient had a Glasgow Coma Scale of 12, with convulsions and a cough with severe respiratory distress. The patient with murine typhus who died had severe diarrhea, progressive impairment of consciousness, and renal failure.

**Figure 3:**
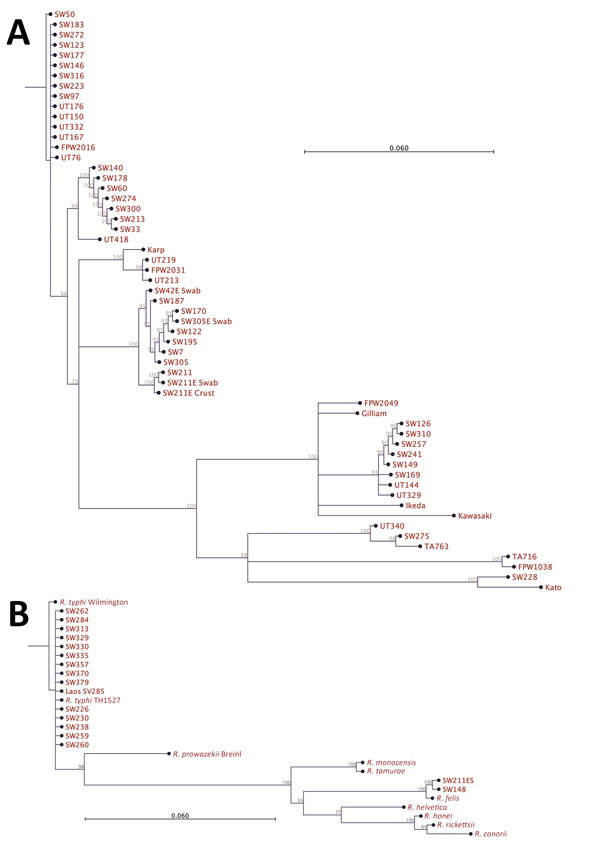
Phylogenetic analysis of pathogens contributing to rickettsial illnesses, Chittagong Medical College Hospital, Chittagong, Bangladesh, August 2014–September 2015. A) Phylogenetic dendrogram based on the nucleotide sequence of the partial open reading frame of the 56-kDa TSA gene (aligned and cropped to ≈450 bp), depicting *Orientia tsutsugamushi* strains in relationship with reference and other strains. *O. tsutsugamushi* genotypes in Bangladesh included Karp, Gilliam, Kato, and TA763 strains, with a predominance of Karp-like strains. B) *Rickettsia* spp. as characterized by 17-kDa gene sequencing (aligned and cropped to 314 bp). The predominant pathogen identified was *R. typhi*. Of note, 2 *R. felis* infection cases were identified, including 1 systemic bloodstream infection and 1 scrub typhus case with eschar swab positivity in patient no. SW211ES (Technical Appendix Table), whose blood specimen was negative for *R. felis*, suggesting possible skin colonization of *R. felis*. Scale bar indicates nucleotide substitutions per site; branches shorter than 0.002 are shown as having a length of 0.002.

Most 56-kDa gene sequences (cropped to ≈450 bp length) clustered with Karp and Karp-like sequences, previously described from Thailand (*12*), but we also observed a cluster of Gilliam-like strains. One sequence (SW275) grouped closely with the Thai animal TA763 strain and another (SW228) with the Kato reference strain (Figure 3). We found no evidence of substantial divergence from known *O. tsutsugamushi* strains or potential new *Orientia* species, and *Orientia chuto* sp. nov. from Dubai remains highly distinct in the 47-kDa gene sequence phylogenetic tree (Technical Appendix Figure) (*13*,*14*). Pairwise gene–gene sequence similarity values between *O. tsutsugamushi* samples from Bangladesh and reference strains (partial 56-kDa gene sequences) showed that the largest proportion of 56-kDa gene sequences were similar to the Karp reference strain or the Thai Karp-like strain UT76, followed by Gilliam (Technical Appendix Table).

Most 17-kDa sequences (cropped to 314 bp) obtained from patient blood showed highest homologies to the *R. typhi* Wilmington strain, the TH1527 Thai strain from Chiangrai, and recently described strains from Laos and Yucatan, Mexico (GenBank accession nos. AE017197, CP003398, KC283066, and JX198507, respectively). Two 17-kDa sequences from SW148 (blood) and SW211ES (eschar swab) showed 100% homologies with the previously sequenced *R. felis* strain URRWXCal2 and a recent isolate from cat fleas on opossums in Yucatan (GenBank accession numbers CP000053 and KR709306, respectively).

## Discussion

Rickettsial illnesses are common causes of fever in hospitalized patients in southeastern Bangladesh. In total, 23% of all undifferentiated febrile case-patients recruited prospectively over a full calendar year were attributable to rickettsial illnesses, predominantly scrub typhus (16.8%) and murine typhus (5.8%). These febrile patients had disease severity that justified hospitalization and an overall mortality rate of 4.3% (18/417). The case-fatality rate for rickettsial illness was high (4% overall) and similar for each group (4.3% [3/70] for scrub typhus, 3.6% [1/28] for murine typhus, and 4.4% [14/320] for non–rickettsial fever).

The nonspecificity of clinical symptoms and lack of readily available accurate diagnostic tools continue to contribute to suboptimal recognition of these common infections. Less than half of the patients with scrub or murine typhus had evidence of positivity in both the PCR and serologic assays, confirming the importance of using both diagnostic modalities. In this study, nearly one quarter of hospitalized, febrile patients had easily treatable typhus cases, justifying consideration of the inclusion of doxycycline as part of the empirical treatment for febrile patients in this region; doxycycline is well-tolerated, inexpensive, and readily available in this setting. This study did not specifically target severe disease or vulnerable populations, but if extrapolation of data from recent reports in neighboring countries is valid, a high mortality rate associated with these groups can be assumed for both scrub and murine typhus. Further investigations in the region are urgently needed (*15*,*16*).

We observed a strong seasonal pattern in incidence, with an increase in scrub typhus cases before and at the end of the rainy season and a decrease in cases in the middle of the dry season, when the temperature fell (Figure 2). Nearly two thirds (64% [45/70]) of all scrub typhus cases occurred during October–December and in April, representing relevant periods of which clinicians should be aware. This biphasic pattern could be associated with reduced exposure by humans because of seasonal variation in their activities, a lower number of infected mites emerging from dry soil, or the transience of immune protection previously observed during multiple reexposures (*17*). The low frequency of eschars observed in scrub typhus patients in this study (4%), coupled with the high seroprevalence observed in the same region, probably reflects the presence of partial immune protection, which suggests continuous exposure in this population (*6*,*17*,*18*). Murine typhus cases peaked in the second half of the dry season, consistent with previously reported seasonality (*19*).

Despite multiple reports of serologic evidence for rickettsial illnesses in Bangladesh, no genotyping or molecular characterization of *Orientia* spp. and *Rickettsia* spp. has been performed to date. This step is an essential prerequisite to the development of rapid diagnostic tests and vaccines in disease-endemic areas. During World War II, the failed attempt to produce a scrub typhus vaccine in cotton rat lungs (termed Operation Tyburn) highlighted the importance of correctly identifying the circulating strains causing human disease. That vaccine, based on a clinically relevant strain identified in troops in the Burma Campaign at Imphal, failed to protect troops when tested in Malaya, where different *Orientia* strains are present, and showed no evidence for cross-protection when tested during a large outbreak of scrub typhus (*20*–*22*).

A large, multicenter, high-quality *Orientia* genotyping effort in India recently reported a predominance of Kato-like strains in the south but a high proportion of Karp-like strains in the north (*2*). In our study, we found a spectrum of diverse *Orientia* strains, most of which were Karp-like strains, with Gilliam strains being common, and few samples representing TA763 and Kato-related *O. tsutsugamushi* strains (Figure 3). The dominance of Karp-like strains along with frequent Gilliam strains matches well with genotypic findings from nearby populations in northeast India, Myanmar, and Thailand (*2*,*3*,*12*). These findings should inform the development of improved and more accurate rapid diagnostic tests, especially those using antigen-based assays (*23*). We saw no evidence for new or highly divergent strains on the basis of homologies of the more conserved 47kDa *htra* gene sequences, and *O. chuto* sp. nov. remains a distinct outlier (*14*; Technical Appendix Figure).

This study also identified a large number of murine typhus cases, and the 17-kDa gene–derived *Rickettsia typhi* DNA sequences were remarkably similar to the Wilmington reference strain, the first human Thai isolate from 1965 in Chiangrai, Thailand (TH1527), and a recent case from Laos (SV285) (*24*). Despite the worldwide coastal distribution of murine typhus, as commonly described in various textbooks, little is known about the molecular epidemiology of this potentially severe disease.

The enigmatic role of *R. felis* in human disease is being unraveled, with emerging evidence indicating both pathogenic and opportunistic roles (*25*,*26*). The atypical symptoms of primary infection attributable to *R. felis*, the potential of mosquitoes as transmission vectors, and *R. felis* as a skin contaminant on healthy persons are intriguing recent findings (*27*,*28*). In our study, the blood-borne *R. felis* (SW148) was associated with an unusual papulo-vesicular skin rash. This finding is consistent with yaaf, a previously described vesicular fever representing primo-infection (*27*). A second case (SW211) in a patient with robust evidence of scrub typhus showed the presence of *R. felis* DNA in the superficial eschar swab only, possibly extending previous findings of *R. felis* as a skin contaminant in Africa to the continent of Asia (*29*). The recent evidence for *R. felis* playing a possible role in human disease and skin colonization in Laos and Bangladesh, along with widespread seroprevalence observed in cats and fleas, provide sufficient evidence to pursue investigations into the transmission of this pathogen and its maintenance in nature (*7*,*9*,*28*,*30*).

Limitations of this study include the fact that patients were enrolled from 1 hospital only, limiting the reports to the catchment area. However, this is a large single-study site, given that it is the main tertiary referral hospital for southern Bangladesh (Figure 1). A multicenter effort would provide a more representative range of genotypes across the country, as previously performed in India (*2*). In addition, being hospitalized, the patients in this study were more likely to have symptoms toward the more severe end of the clinical spectrum.

In summary, scrub and murine typhus are important infectious diseases contributing substantially to the burden of undifferentiated fever in Bangladesh. With a mortality rate of 4% each, these diseases clearly require more attention. Empiric treatment strategies should be adapted to cover these treatable rickettsial illnesses, and awareness among medical staff should be promoted regarding the diagnostic difficulties and seasonality of acute febrile illnesses. Studies assessing the prevalence of rickettsial illnesses should use both PCR and serologic testing to avoid missing cases and should cover 1 full calendar year to identify and adjust for seasonality. Further studies focusing on more in-depth assessment of transmission, incidence, and potential impact of these illnesses in Bangladesh are needed to increase awareness, improve empiric treatment strategies, and inform public health interventions aiming to reduce exposure.

Technical Appendix Pairwise gene-gene sequence similarity values between Bangladesh *O. tsutsugamushi* and reference strains. Phylogenetic tree constructed by using the 47-kDa sequences obtained from clinical samples in this study.
