# Supplementary material for: Rickettsial Illnesses as Important Causes of Febrile Illness in Chittagong, Bangladesh
Source: Emerg Infect Dis. 2018 Apr;24(4):638–45. doi: 10.3201/eid2404.170190 (PMC5875266; doi:10.3201/eid2404.170190)
Supplement: Technical Appendix — Pairwise gene-gene sequence similarity values between Bangladesh O. tsutsugamushi and reference strains. Phylogenetic tree constructed by using the 47-kDa sequences obtained from clinical samples in this study. [file 17-0190-Techapp-s1.pdf]

Article DOI: https://doi.org/10.3201/eid2404.170190

Rickettsial Illnesses as Important Causes of Febrile Illness in Chittagong, Bangladesh

Technical Appendix

Technical Appendix Table. Pairwise gene-gene sequence similarity values between Bangladesh *O. tsutsugamushi* and reference strains

Karp	psv *	Gilliam	psv	Kato	psv	TA763	psv	TA716	psv	Kawasaki	psv	Thai UT76	psv
SW122	0.05	SW169	0.03	SW228	0.06	SW275	0.02	SW275	0.20	SW169	0.12	SW223	0.00
SW170	0.05	SW126	0.04	SW123	0.25	SW123	0.22	SW228	0.25	SW126	0.13	SW316	0.00
SW187	0.05	SW149	0.04	SW146	0.25	SW146	0.22	SW123	0.28	SW149	0.13	SW97	0.00
SW195	0.05	SW241	0.04	SW177	0.25	SW177	0.22	SW146	0.28	SW241	0.13	SW123	0.00
SW305	0.05	SW257	0.04	SW183	0.25	SW183	0.22	SW177	0.28	SW257	0.13	SW146	0.00
SW305ES	0.05	SW310	0.04	SW223	0.25	SW223	0.22	SW183	0.28	SW310	0.13	SW177	0.00
SW42ES	0.05	SW140	0.18	SW272	0.25	SW272	0.22	SW223	0.28	SW228	0.24	SW183	0.00
SW7	0.05	SW178	0.18	SW316	0.25	SW316	0.22	SW272	0.28	SW123	0.24	SW272	0.00
SW211	0.05	SW213	0.18	SW50	0.25	SW50	0.22	SW316	0.28	SW146	0.24	SW50	0.00
SW211EC	0.05	SW274	0.18	SW97	0.26	SW97	0.22	SW50	0.28	SW177	0.24	SW140	0.03
SW211ES	0.05	SW300	0.18	SW140	0.26	SW211	0.23	SW97	0.28	SW183	0.24	SW178	0.03
SW123	0.05	SW33	0.18	SW178	0.26	SW211EC	0.23	SW122	0.30	SW223	0.24	SW213	0.03
SW146	0.05	SW60	0.18	SW213	0.26	SW211ES	0.23	SW170	0.30	SW272	0.24	SW274	0.03
SW177	0.05	SW123	0.18	SW274	0.26	SW122	0.23	SW187	0.30	SW316	0.24	SW300	0.03
SW183	0.05	SW146	0.18	SW300	0.26	SW170	0.23	SW195	0.30	SW50	0.24	SW33	0.03
SW223	0.05	SW177	0.18	SW33	0.26	SW187	0.23	SW305	0.30	SW97	0.24	SW60	0.03
SW272	0.05	SW183	0.18	SW60	0.26	SW195	0.23	SW305ES	0.30	SW140	0.25	SW122	0.06
SW316	0.05	SW223	0.18	SW169	0.26	SW305	0.23	SW42ES	0.30	SW178	0.25	SW170	0.06
SW50	0.05	SW272	0.18	SW126	0.27	SW305ES	0.23	SW7	0.30	SW213	0.25	SW187	0.06
SW97	0.06	SW316	0.18	SW149	0.27	SW42ES	0.23	SW140	0.30	SW274	0.25	SW195	0.06
SW140	0.06	SW50	0.18	SW241	0.27	SW7	0.23	SW178	0.30	SW300	0.25	SW305	0.06
SW178	0.06	SW97	0.19	SW257	0.27	SW140	0.24	SW213	0.30	SW33	0.25	SW305ES	0.06
SW213	0.06	SW211	0.19	SW310	0.27	SW178	0.24	SW274	0.30	SW60	0.25	SW42ES	0.06
SW274	0.06	SW211EC	0.19	SW211	0.28	SW213	0.24	SW300	0.30	SW211	0.25	SW7	0.06
SW300	0.06	SW211ES	0.19	SW211EC	0.28	SW274	0.24	SW33	0.30	SW211EC	0.25	SW211	0.07
SW33	0.06	SW122	0.19	SW211ES	0.28	SW300	0.24	SW60	0.30	SW211ES	0.25	SW211EC	0.07
SW60	0.06	SW170	0.19	SW122	0.28	SW33	0.24	SW211	0.30	SW275	0.26	SW211ES	0.07
SW169	0.21	SW187	0.19	SW170	0.28	SW60	0.24	SW211EC	0.30	SW122	0.26	SW126	0.20
SW275	0.21	SW195	0.19	SW187	0.28	SW126	0.26	SW211ES	0.30	SW170	0.26	SW149	0.20
SW126	0.21	SW305	0.19	SW195	0.28	SW149	0.26	SW169	0.34	SW187	0.26	SW241	0.20
SW149	0.21	SW305ES	0.19	SW305	0.28	SW241	0.26	SW126	0.34	SW195	0.26	SW257	0.20
SW241	0.21	SW42ES	0.19	SW305ES	0.28	SW257	0.26	SW149	0.34	SW305	0.26	SW310	0.20
SW257	0.21	SW7	0.19	SW42ES	0.28	SW310	0.26	SW241	0.34	SW305ES	0.26	SW169	0.21
SW310	0.21	SW228	0.19	SW7	0.28	SW169	0.26	SW257	0.34	SW42ES	0.26	SW275	0.21
SW228	0.24	SW275	0.22	SW275	0.31	SW228	0.29	SW310	0.34	SW7	0.26	SW228	0.23

This matrix presents pairwise gene-gene sequence similarity values between *O. tsutsugamushi* samples from Bangladesh and reference strains (partial 56kDa gene sequences). Grading: highest nucleotide sequence similarities (top) to lowest (bottom). Similar sequences with values <0.1 are shaded in gray. The largest proportion of 56kDa gene sequences were similar to the Karp strain, or Karp-like strain (UT76). ES = Eschar swab, EC = eschar crust sample specimens. Calculations performed using MEGA version 7 software. Note: * psv = these values represent pairwise gene-gene sequence similarity values ranging from 0 (genetically identical) – to 1 (genetically different).

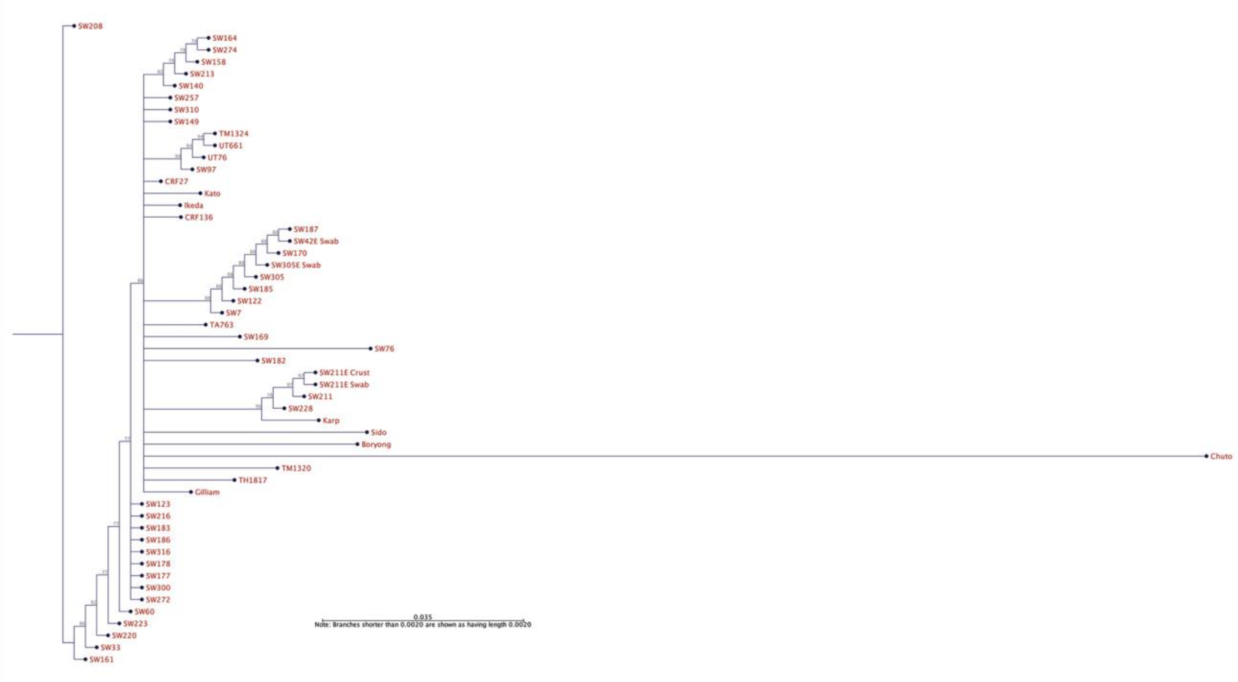

Technical Appendix Figure. Phylogenetic tree constructed using the 47-kDa sequences obtained from clinical samples in this study. There was no evidence for new or highly divergent strains based on homologies of the more conserved 47kDa *htra* gene sequences, and *O. chuto* sp. nov. remains a distinct outlier. The scale bar length segment with the number 0.035 shows the length of branch that represents that amount of genetic change (i.e., 3.5% or 7 nucleotides change/200 nucleotide sites); branches shorter than 0.002 are shown as having a length of 0.002.
